# Environmentally Friendly Layered Double Hydroxide Conversion Layers: Formation Kinetics on Zn–Al–Mg-Coated Steel

**DOI:** 10.1021/acsami.1c19573

**Published:** 2022-01-20

**Authors:** Tobias Holzner, Gerald Luckeneder, Bernhard Strauß, Markus Valtiner

**Affiliations:** † voestalpine Stahl GmbH, Research and Development, voestalpine Str. 3, 4020 Linz, Austria; ‡ Vienna University of Technology, Institute for Applied Physics, A-1040 Vienna, Austria

**Keywords:** LDH, Zn−Al−Mg-coated steel, conversion layer, formation kinetics, in situ OCP, cyclic voltammetry

## Abstract

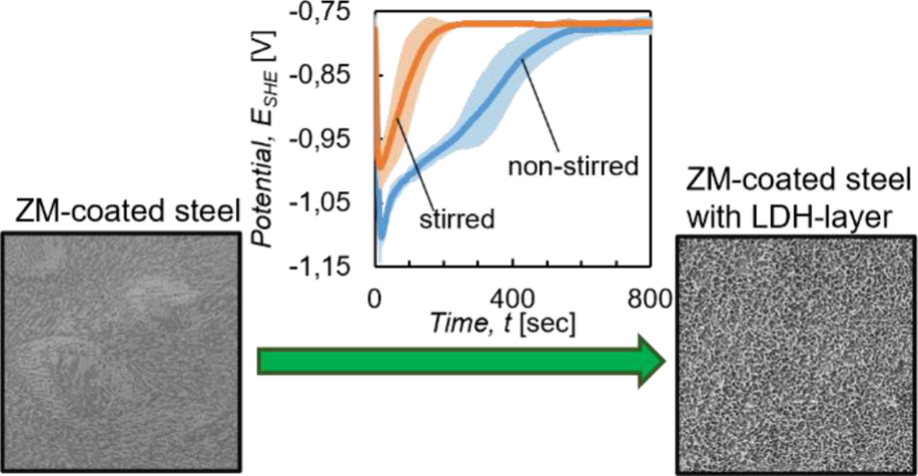

Phosphate- or chromate-based industrially produced conversion layers, while effectively increasing adhesion for organic coatings and corrosion resistance, come at the cost of environmentally problematic and harmful treatment solutions and waste. In this respect, layered double hydroxide (LDH)-based conversion layers offer an environmentally benign alternative without toxicologically concerning compounds in the treatment solution. Here, we study an LDH conversion layer on Zn–Al–Mg-coated steel (ZM-coated steel), which was produced by immersion into a carbonate- and magnesium-containing alkaline solution. The mechanism and kinetics of the conversion layer formation were investigated with in situ open circuit potential measurements, cyclic voltammetry (CV), and scanning electron microscopy (SEM). Acceleration of the LDH layer formation through high convection in the treatment solution was found. This was attributed to a higher oxygen availability at the metal/solution interface because no diffusion-limited state during the layer formation is reached due to high convection. The importance of oxygen within the kinetics indicates a corrosion-like mechanism, with cathodic and anodic sites on the steel sample. The LDH formation happens by co-precipitation of ions present in the treatment solution and dissolved ions from the ZM-coated steel. With CV, SEM, and X-ray diffraction, the growth of the LDH conversion layer was investigated with respect to the immersion time. It was found that after 30 s, the sample surface was almost fully covered with an LDH layer, and with the increasing immersion time, the layer grows in thickness. Increased understanding on the kinetics and mechanism of the LDH conversion layer formation on ZM-coated steel gives rise to a targeted optimization of the treatment solution and process parameters.

## 1 Introduction

Conversion layers are synthesized on metal substrates like steel or hot dip-galvanized steel to increase the corrosion persistence and to provide better adhesion between the substrate and organic coatings or paints, which may be applied afterward.^1^ Conversion layers may also act as forming aids and therefore improve the formability of metals.^2,3^ Industrially produced conversion layers are often based on phosphates or chromates. Phosphate conversion layers consist of tertiary metal phosphates [e.g., Zn_3_(PO_4_)_2_] and are widely but not exclusively applied in the automotive industry prior to painting.^2,4,5^ Typically, a tri-cation phosphating process containing Zn, Ni, and Mn is used in the automotive sector, resulting in the deposition of Zn_3–*x*–*z*_(Ni_*x*_, Mn_*z*_)(PO_4_)_2_ for increased paint adhesion and corrosion protection.^6^ However, phosphate-based conversion layers come with harmful components in the phosphating treatment solution like Ni or nitrite and environmental problematic waste products.^2,5,7^ Chromate-based conversion layers are typically formed using hexavalent chromium containing solutions and provide high corrosion protection on different materials like aluminum, magnesium, or galvanized steel.^8−12^ However, Cr(VI) is known for its high carcinogenic risk and overall harmful impact on human health, which led to strict legal restrictions, especially in the European Union since 2017.

The mentioned issues of the widely used phosphate- or chromate-based conversion layers account for the importance of the research on developing new conversion layer treatments for several metal substrates, like Al, Mg, and Zn, in the last decade.^2,13^ Those alternative conversion layers are often but not exclusively based on silanes, molybdate, cerium, vanadate, or zirconium.^13^ For Zn–Al–Mg-coated steel, zirconium-based conversion layers are reported in previous work by Lostak et al.^14^ or Han et al.,^15^ who investigated the formation mechanism and acceleration effects of NO_3_^–^ and Cu(II) in the treatment solution. Buchheit et al.^16^ successfully synthesized Al–Zn layered double hydroxide (LDH) layers on galvanized steel and found that an immersion time of 10 min into the treatment solution is necessary to obtain uniform coatings. Nevertheless, no work on LDH conversion layers on Zn–Al–Mg-coated steel is reported yet.

LDHs are compounds with the general chemical formula [M^2+^_1–*x*_M^3+^_*x*_(OH)_2_]^*x*+^[A^*n*–^]_*x*/*n*_·*z*H_2_O, where M^2+^ and M^3+^ are divalent and trivalent metal cations and A^*n*–^ are inorganic or organic anions. LDHs are known as anion exchange materials because the anion in the layered structure can be substituted easily. This property makes LDH compounds highly interesting for many different possible applications, like utilizing them as nanocontainers for drug delivery.^17,18^ LDH compounds also exhibit promising anticorrosive properties that were proven for substrates like Al and Mg alloys in many studies. The physical barrier effect^19^ and chloride binding effect through anion exchange^20−22^ are the most often stated corrosion protection mechanisms of LDHs. In situ synthesized LDH layers or LDH particles dispersed in organic coatings are typically used for corrosion protection. LDH particles can act as anticorrosion particles themselves via anion exchange or act as nanocontainers for corrosion inhibitors, which are intercalated as the anion in the LDH structure.^22,23^

In situ synthesized LDH layers are synthesized by immersion of metallic substrates into solutions containing none,^24^ one,^25^ or both^26^ metal cations, of which the LDH layer is supposed to be made of. In 1994, Buchheit et al.^27^ fabricated Li–Al–CO_3_ LDH films on Al substrates by immersion into an alkaline Li_2_CO_3_ solution and additionally demonstrated an increased corrosion resistance of Al due to the LDH layer. The anion can be incorporated into the LDH compound from the treatment solution either directly or by a second anion exchange step after the formation of the LDH layer. Tedim et al.^28^ prepared Zn–Al–NO_3_ LDH conversion layer on Al substrates, and in the second reaction step, they changed the NO_3_ anion with V_2_O_7_ by immersion into a NaVO_3_ solution and thereby increased the corrosion protection property of the LDH layer. Anjum et al.^29^ successfully synthesized Mg–Al LDH layers on Mg alloy with different intercalated corrosion inhibitors (8HQ, SB, and APTS) by a single step immersion process and demonstrated the corrosion protection of the Mg alloy as well.

Recently, Bouali et al.^30^ investigated the mechanism of Zn–Al–NO_3_ LDH conversion layer formation on Al alloy and proposed a three-stage mechanism. First, the native oxide layer is converted into an AlOOH intermediate layer, which partially dissolves in the second stage because of an increase of the local pH, leading to the growth of first LDH crystals. In the last stage, the LDH conversion layer grows in thickness, resulting in a dense and covering layer. Mikhailau et al.,^31^ on the other hand, investigated the mechanism of Zn–Al–NO_3_ LDH conversion layer formation on zinc. They also found a three-stage mechanism with nitrate reduction and zinc oxidation in the first stage, where OH^–^ from the nitrate reduction is buffered by the aluminum in the treatment solution. In Stage 2, aluminum hydroxide precipitates on the Zn substrate. In the last stage, the presence of aluminum and zinc hydroxide leads to the formation of the LDH layer on the Zn substrate.

This study aims to investigate the mechanism and kinetics of, to our knowledge, the unique LDH conversion layer formation on ZM-coated steel. A one-step immersion process using a carbonate- and magnesium-containing alkaline treatment solution is used for the conversion layer formation. Because the treatment solution contains no toxicological or environmental concerning compounds, LDH conversion layers offer an environmentally benign alternative without major toxicity concerns. The influence of the LDH conversion layer on the corrosion resistance of ZM-coated steel, especially under chloride containing conditions, will be covered in detail in a subsequent article.

## 2 Experimental Section

### 2.1 Materials

**Figure 1:**
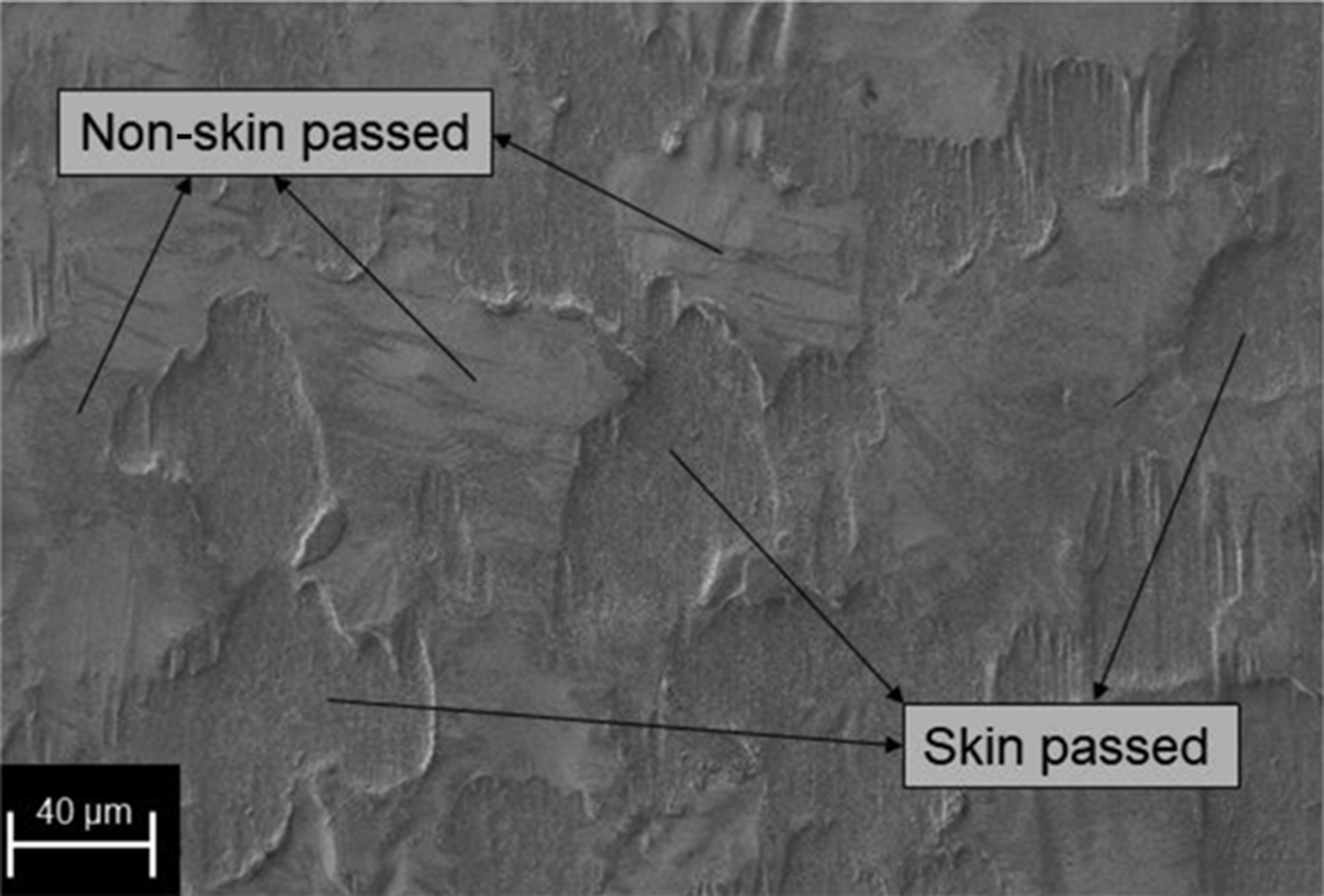
Surface of a skin pass-rolled ZM-coated steel sample.

In this study, we used a Zn–Al–Mg coated steel sheet (ZM-coated), which was produced by hot dip galvanizing with a Zn–Al–Mg alloy at an industrial production plant and was provided by voestalpine Stahl GmbH (Linz, Austria). Chemical composition and coating weight are listed in Table 1. Additionally, the ZM-coated steel sheets are skin pass-rolled, leading to two different surface conditions on a microstructural scale, namely, skin passed and non-skin passed areas. Figure 1 shows an SEM image of the surface of a skin pass-rolled ZM-coated steel sample.

**Table 1:** Composition and Weight of the ZM Coating of the ZM-Coated Steel Sheets

label	Zn (%)	Al (%)	Mg (%)	coating weight (g/m^2^)	steel thickness (mm)
ZM90MC	96	2.5	1.5	90	0.75

### 2.2 Conversion Layer Synthesis

In a first step, we cleaned the ZM-coated steel sheets with an aqueous alkaline cleaning solution, containing 10 g/L *Bonderite C-AK C 72* (Henkel AG & Co. KGaA) that was heated to 40 °C. Following the cleaning step, the steel sheets were rinsed with deionized water. These steps are repeated one more time, and finally, the cleaned steel sheets are dried with hot air.

For the preparation of the LDH conversion layer, we immersed the steel sheets into the LDH treatment solution that was heated to 80 °C. We varied the immersion time from 5 to 300 s and, depending on the experiment, the solution was stirred or not stirred. The treatment solution contains 3 mmoL/L CO_3_^2–^ and 5 mmoL/L Mg^2+^ and has a pH of 10.5. Subsequent to the immersion step, we thoroughly rinsed the samples with deionized water and dried them with compressed air.

Two modified treatment solutions concerning the oxygen concentration were used for certain experiments, namely, an O_2_-enriched and O_2_-depleted solution. The O_2_-enriched solution was prepared by bubbling O_2_ gas into the treatment solution for 5 min directly before the ZM-coated steel sample was immersed. For the O_2_-depleted solution, Ar gas was bubbled into the solution for 5 min directly before the sample was immersed after the solution was put in an ultrasonically bath for 10 min.

### 2.3 Electrochemical Measurements

Electrochemical measurements were performed using a Metrohm Multi Autolab Cabinet potentiostat/galvanostat.

For in situ open circuit potential (OCP) measurements, 2 × 10 cm ZM-coated steel samples (working electrode) and a Ag/AgCl reference electrode (3 M KCl) were immersed into the stirred or not stirred LDH treatment solution. The measurement was started immediately after the electrodes were fully immersed. The upper side of the working electrode at the solution/air interface was covered with adhesive tape. For each measurement, the same beaker with the same magnetic stirring rod was used to ensure convection conditions as comparable as possible for the measurements in stirred solution.

Cyclic voltammetry (CV) was performed in a three-electrode setup in a cylindrical polycarbonate cell with a platinum foil as a counter electrode and a Ag/AgCl reference electrode (3 M KCl, in a Luggin capillary). The samples were connected as the working electrode with an exposed area of 1 cm^2^. Measurements were carried out in a quiescent borate-sulfate-boric acid buffer electrolyte (0.05 M Na_2_B_4_O_7_, 0.05 M Na_2_SO_4_, and 0.2 M H_3_BO_3_; pH 8.3) and were started after an initial 60 s equilibration time. Cyclic voltammograms were recorded within a voltage range of −0.55 to −1.20 V_SHE_ (starting potential 0 V_OCP_) with a scan rate of 100 mV/s at room temperature. Eight cycles were measured consecutively, and the last one was used for evaluation.

By integrating the reaction peaks of the cyclic voltammograms in the current density vs time plot, the charge, which is converted during the o*x*idation and reduction reaction, was calculated. Considering the Pourbaix diagrams of Zn, Al, and Mg and the literature,^32^ the redox reaction that occurs during the CV measurement can be attributed to the Zn oxidation and reduction of the oxidized species.

Losch et al. established a method to determine the coverage of phosphate layers with CV measurements.^33^ This method was already used to analyze the coverage of different types of layers on a variety of substrates like zinc-coated steel.^32,34,35^ Similar to this method, the coverage of ZM-coated steel samples by the LDH conversion layer was calculated. For evaluation, the charge converted during the oxidation reaction of the eighth cycle was selected. We assume that the Zn distribution of the ZM-coated steel sheets is homogenies on a macroscopic scale because actually the coverage of the free Zn surface of the ZM-coated steel samples is determined with this method. Furthermore, we assumed that the coverage of the Zn-containing phases is representative of the coverage of the whole sample because the ZM coating consists of 96 wt % Zn (Zn dendrites and ternary eutectic Zn/MgZn_2_/Al in equal shares).

### 2.4 Characterization

Surface and cross-section images of the samples were obtained with field emission SEM (Zeiss/Ultra 55). Cross-section samples were prepared by cross-section-polishing using an Argon beam. X-ray diffraction (XRD, PANalytcal X’Pert PRO MPD) measurements were performed at a glancing angle of 3° using a Cobalt Kα radiation (λ = 1.78 Å) at a scanning rate of 1.5°/min in the 2θ range of 10–115°.

Polarization modulation infrared reflection absorption spectroscopy (PM-IRRAS) was performed with a Vertex 70v infrared spectrometer with a PMA50XL module and an LN-MCT detector. PM-IRRAS spectra were recorded at an incident angle of 78° with a scan rate of 4 cm^–1^. The background for each measurement was air, and the spectrum of ZM-coated steel without the LDH layer was used as reference. Therefore, the presented PM-IRRAS spectra show the changes in the spectrum due to the LDH treatment. X-ray photoelectron spectroscopy (XPS) was performed using a Thetaprobe XPS system with a monochromated Al Kα X-Ray source (1486.6 eV). Survey spectra were measured using a pass energy of 200 eV and energy step rate of 1 eV. For high-resolution spectra, 50 eV pass energy and 0.05 eV step rate were used. Charge shifting was corrected using the C 1s peak of adventitious carbon at a binding energy of 185.0 eV. Chemical assessment was performed by comparison of the modified auger parameters of the measured element signals with reference materials measured on the same XPS device under the same experimental conditions done by Duchoslav et al.^36−38^

### 2.5 Corrosion Tests

A neutral salt spray (NSS) test was performed, according to ISO 9227:2017, with ZM-coated steel sheets with and without an LDH layer. In addition, ZM-coated steel with LDH and phosphate-based conversion layers were tested in a cyclic corrosion test, according to VDA 233-102 (DIN 55635:2019), with a glass plate on top of the samples (120 μm gap between glass and sample) to simulate flange conditions, as described in SEP 1160.^39^

## 3 Results and Discussion

### 3.1 Characterization of In Situ Synthesized LDH Conversion Layers on Zn–Al–Mg-Coated Steel

In Figure 3a, the XRD pattern in the 2θ range of 10–115° of ZM-coated steel is shown before and after the immersion into the stirred LDH treatment solution for 300 s. One can see that the two patterns only differ in one reflex appearing after the LDH treatment at around 12.7°. By comparison with XRD databases (COD, ICSD, and ICDD), zinc, aluminum, MgZn_2_, and the LDH compound Mg_0.833_Al_0.167_(OH)_2_(CO_3_)_0.083_·0.75H_2_O were found in the LDH-treated sample. Zinc, aluminum, and MgZn_2_ are measured from the ZM coating, indicating that the present LDH conversion layer is either not fully covering the surface or is a rather a thin layer. The reflex at 12.7° was assigned to the LDH compound, which represents the 003 basal plane. *d*(003) is 7.9 Å and is in good agreement with literature.^40−42^ All other reflexes of the Mg–Al–CO_3_ LDH compound are overlapped by the much more intense reflexes of the other phases present in the sample. Therefore, only the 12.7° reflex can be used to verify the existence of an LDH conversion layer on ZM-coated steel. It should be noted that small changes in the exact stoichiometric composition of the LDH layer, for example, different M^2+^/M^3+^ ratios or small amounts of Zn incorporated in the LDH compound, lead to only small shifts of the reflex.^43,44^ It may not be ruled out that the exact stoichiometric composition of the synthesized LDH conversion layer may locally differ slightly from the found Mg_0.833_Al_0.167_(OH)_2_(CO_3_)_0.083_·0.75H_2_O, especially when it comes to the M^2+^/M^3+^ ratio. Trace amounts of Zn–Al–CO_3_ LDH or Mg/Zn–Al–CO_3_ LDH compounds may also be present but not be significantly measureable using XRD because of the overlapping with the much more intense reflexes of the underlying ZM coating and due to limitations of database entries.

**Figure 2:**
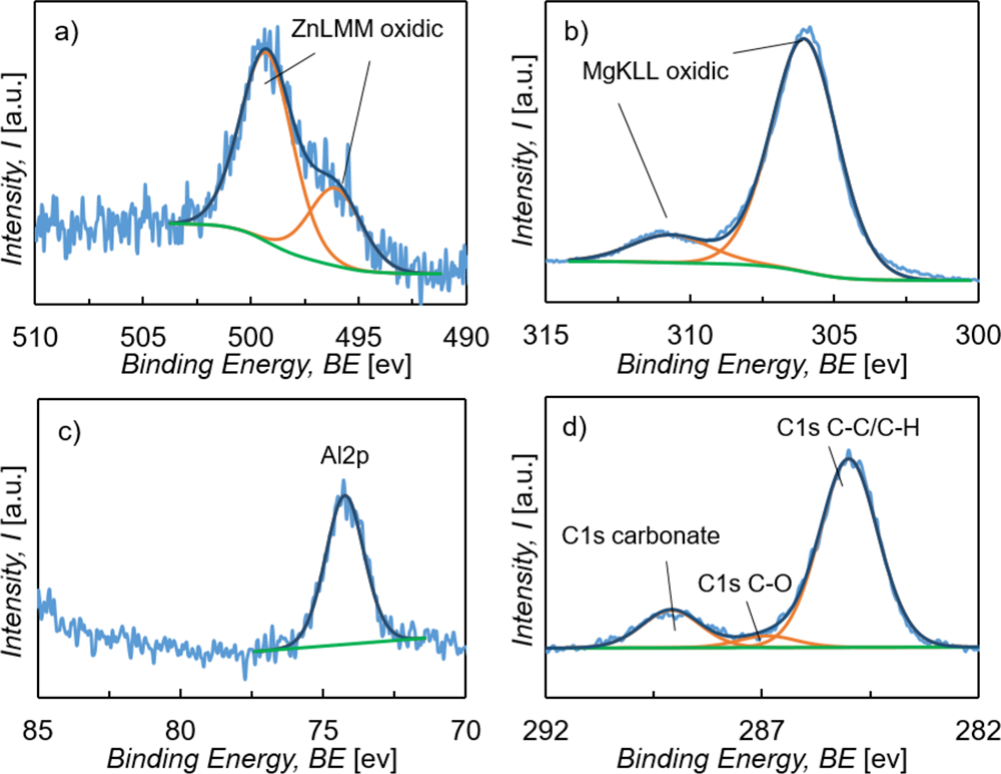
High-resolution XPS spectra of ZM-coated steel after immersion into the LDH treatment solution for 5 min of (a) Zn LMM, (b) MgKLL, (c) Al 2p, and (d) C 1s.

For further characterization of the composition of the prepared LDH conversion layer, XPS measurements of three parallel samples after 5 min immersion into the stirred treatment solution were performed. The elemental composition gained from XPS survey spectra is listed in Table 2, and in Figure 2, high-resolution XPS spectra of the selected signals of Zn (a), Mg (b), Al (c), and C (d) are shown from one sample. The survey spectra and high-resolution spectra of other elements were recorded but are not presented in this work.

**Table 2:** Elemental Composition of the LDH Layer Formed on ZM-Coated Steel Samples after 5 min Immersion into the Treatment Solution^a^

	concentration (at%)			
peak	sample 1	sample 2	sample 3	average
O 1s	54.2	53.0	57.1	54.8 ± 2.1
Mg 2p	19.2	19.8	19.4	19.4 ± 0.3
C 1s	19.6	19.6	13.6	17.6 ± 3.4
Al 2p	3.8	3.7	4.8	4.1 ± 0.6
Zn 2p3	1.7	2.0	1.9	1.8 ± 0.2
S 2p	0.0	0.0	1.4	0.5 ± 0.8
Ar 2p	0.8	1.4	1.3	1.2 ± 0.3
Cl 2p	0.7	0.5	0.5	0.6 ± 0.1

a Calculated from XPS survey spectra. Three parallel samples were analyzed.

With an Mg/Al ratio of 4.8:1, the XPS results are in good agreement with the XRD measurement, which revealed a Mg–Al–LDH compound with a Mg/Al ratio of 5:1. In addition, the XPS results show that there is indeed a small amount of Zn present, and by comparing the modified auger parameter of the Zn signal, obtained by individual high-resolution XPS spectra, with reference materials, the Zn signal was assigned to a Zn/Mg–Al LDH compound. In Figure 2d, one can see that there are three different C-signals, whereby the C–O and C–C/C–H signals originate from adventitious carbon impurities. The third signal can be assigned to carbonate ions, indicating the presence of carbonate anions incorporated in the LDH layer.

**Figure 3:**
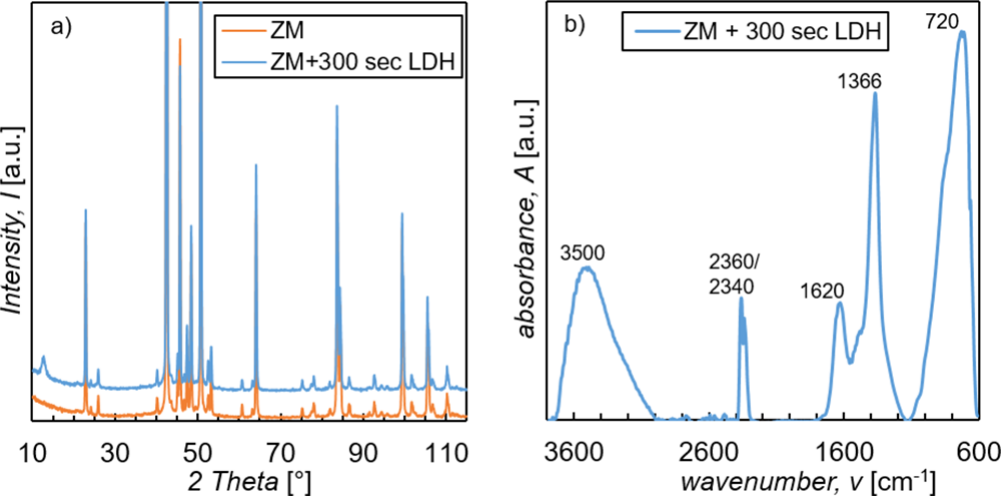
(a) XRD patterns of ZM-coated steel (orange) and ZM-coated steel after immersion into the stirred treatment solution for 300 s (blue). (b) PM-IRRAS spectrum of ZM-coated steel after immersion into the stirred treatment solution for 300 s referenced to ZM-coated steel (spectrum shows the difference between ZM-coated steel with and without an LDH layer)

Figure 3b shows a PM-IRRAS spectrum of ZM-coated steel after the immersion into the stirred LDH treatment solution for 300 s. The spectrum is referenced to ZM-coated steel and therefore shows the changes at the surface trough the LDH treatment. The broad band at 3000–3600 cm^–1^ can be attributed to OH-stretching vibrations from water and metal hydroxides. The two bands present close together at 2340 and 2360 cm^–1^ derive from CO_2_ in the air. At 1620 cm^–1^, the bending vibration of interlayer water molecules in the LDH compound is present. The intense absorption band at 1366 cm^–1^ and the small shoulder at around 1400 cm^–1^ are assigned to the asymmetric CO_3_^2–^ stretching vibration in LDH compounds. Therefore, XRD and PM-IRRAS both indicate that the anion in the LDH conversion layer is CO_3_^2–^. In the region of 600–1000 cm^–1^, a broad and intense band is present, which results from different metal hydroxide translation or deformation vibrations.^40−43,45,46^

XRD, XPS, and PM-IRRAS measurements indicate that by immersion of ZM-coated steel into the LDH treatment solution, a Mg–Al–CO_3_ LDH conversion layer with some Mg^2+^ being substituted with Zn^2+^ is formed. According to XRD and XPS, the Mg/Al ratio is around 5:1.

### 3.2 Influence of Convection in the Treatment Solution on the LDH Conversion Layer Formation

#### 3.2.1 In Situ OCP Measurements

We measured the OCP of ZM-coated steel as a function of time, while the steel sample was immersed into the LDH treatment solution. The formation of conversion layers includes oxidation and reduction reactions at the sample surface, which result in a change of the measurable mixed potential. Therefore, in situ OCP measurements are an appropriate technique to monitor the conversion layer formation and gather information about the reaction/growth mechanism and the formation kinetics.^47−51^

**Figure 4:**
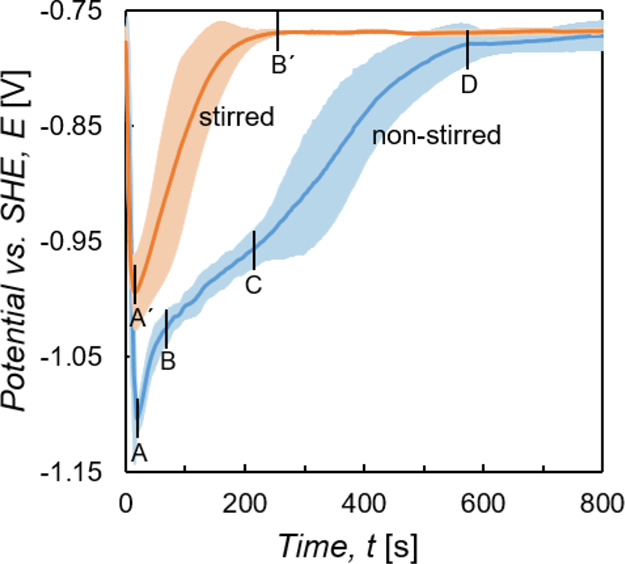
In situ OCP curves of ZM-coated steel immersed into the stirred (orange) and nonstirred (blue) treatment solution. Average of four measurements with standard deviation.

Figure 4 shows the in situ OCP curves of ZM-coated steel in the stirred and nonstirred treatment solution. The graphs represent the average of four measurements each with the corresponding standard deviations.

The in situ OCP curve measured in the nonstirred solution can be divided into four different stages until a steady state is reached, indicated by a constant potential. In Stage 1 (Start → Point A), the potential rapidly drops from −0.78 V_SHE_ to around −1.1 V_SHE_ in the first 20 s. During Stage 2 (Point A → Point B), the potential increases again until reaching Point B, where the potential rise flattens and the OCP curve transitions into the plateau-like Stage 3. After around 200 s (Point C), Stage 4 starts, marked by an increase of the slope once again, until the potential stabilizes at ca. −0.78 V_SHE_ after roughly 550 s immersion time (Point D).

In contrast, the in situ OCP curve measured in the stirred treatment solution consists only of two stages. Stage 1′ is again a quick drop of the potential from −0.78 V_SHE_ to around −1.0 V_SHE_ during the first 10–15 s of immersion time. Then, during Stage 2′ (Point A′ → Point B′), the potential steeply increases again until it reaches a constant value of around −0.78 V_SHE_ after an immersion time of roughly 250 s.

The initial drop of the potential (Stage 1 or 1′) in both stirred and nonstirred conditions can be ascribed to the dissolution of oxides/hydroxides. Similar effects are reported for conversion layer formation on aluminum in alkaline and chloride containing solutions^52,53^ or fluoride containing treatment solutions.^49,51,54^

The following rise of the potential (Stage 2 or 2′) indicates that cathodic reactions get dominant and the sample is passivated through the formation of a conversion layer. When a constant OCP value is measured, steady state between the formed LDH conversion layer and the treatment solution is reached. This indicates that the conversion layer formation is completed, although an increase of film thickness with increasing immersion times cannot be ruled out.^54^

The fact that the plateau-like stage in the OCP curve vanishes, when the treatment solution is stirred, shows that a diffusion-controlled process seems to be involved. Therefore, during Stage 2 in nonstirred solution, the reactants at the metal/solution interface are consumed quickly due to the layer formation. This leads to a depletion at the interface of at least one reactant, likely one originating from the treatment solution. Because of the missing convection in the solution, the diffusion-controlled layer at the metal/solution interface is thick, and consequently, it takes a long time for the depleted reactant to diffuse from the bulk solution to the interface. Thus, during Stage 3, the conversion layer formation is slowed down because of diffusion limitation of at least one of the involved reactions. After a certain amount of time, a constant and sufficient diffusion flux is reached, resulting in an increase of the conversion layer formation rate again. Such non-equilibrium situations during reactive interface changes like in Stage 3 are already known to create time lags in electron transfer, redox reactions, and solution side reactions.^55^

In stirred solution, this diffusion limitation does not exist because the high convection results in a thin diffusion-controlled layer and an overall more uniform distribution of the reactants in the solution. Therefore, one can observe an acceleration of the LDH conversion layer formation.

#### 3.2.2 CV Measurements

**Figure 5:**
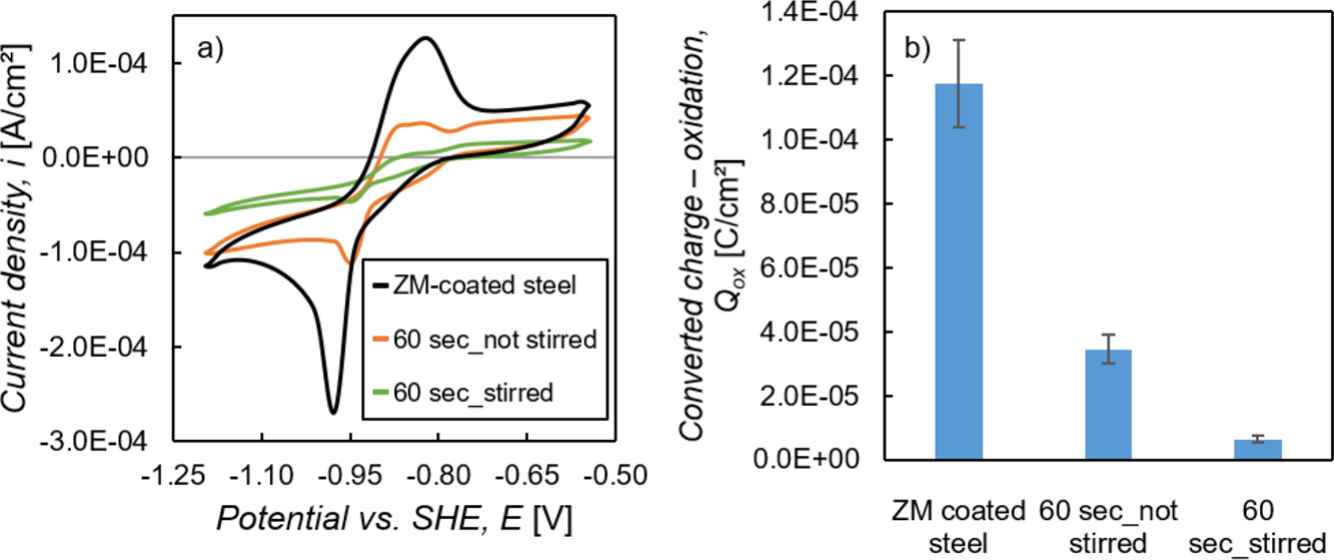
(a) Cyclic voltammograms (8th cycle) of samples immersed for 60 s into the stirred and nonstirred treatment solution. Cleaned ZM-coated steel as reference. (b) Converted charge during the oxidation reaction of the eighth cycle of the CV measurement. Average of five measurements with standard deviation.

With CV measurements, we compared different samples in terms of their electrochemically active free surface of the ZM-coated steel, as described in Section 2.3. In Figure 5a, one can see the cyclic voltammograms of samples that were immersed 60 s in the stirred and nonstirred treatment solution in comparison with a reference sample (cleaned ZM-coated steel). The peaks of both redox reactions as well as the current densities in the “passive region” decrease after immersion of the samples into the treatment solution. This indicates, at least partially, a coverage of the sample surface due to the formation of an LDH conversion layer. The sample immersed in the stirred solution clearly shows less electrochemical activity compared to the one that was treated in nonstirred solution. The charge that is converted during the oxidation reaction of the CV measurement *Q*_ox_ was calculated (see Section 2.3) and is plotted for each of the three samples in Figure 5b. The immersion of ZM-coated steel in the stirred and nonstirred treatment solution for 60 s results in a reduction of *Q*_ox_ by 95 and 71%, respectively. This equals a decrease of *Q*_ox_ of 82% due to the stirring of the solution compared to the nonstirred solution.

These results show that an increased surface coverage by the LDH layer is reached when the treatment solution is stirred and therefore suit the results from the in situ OCP measurements shown in Section 3.2.1.

#### 3.2.3 SEM Images

**Figure 6:**
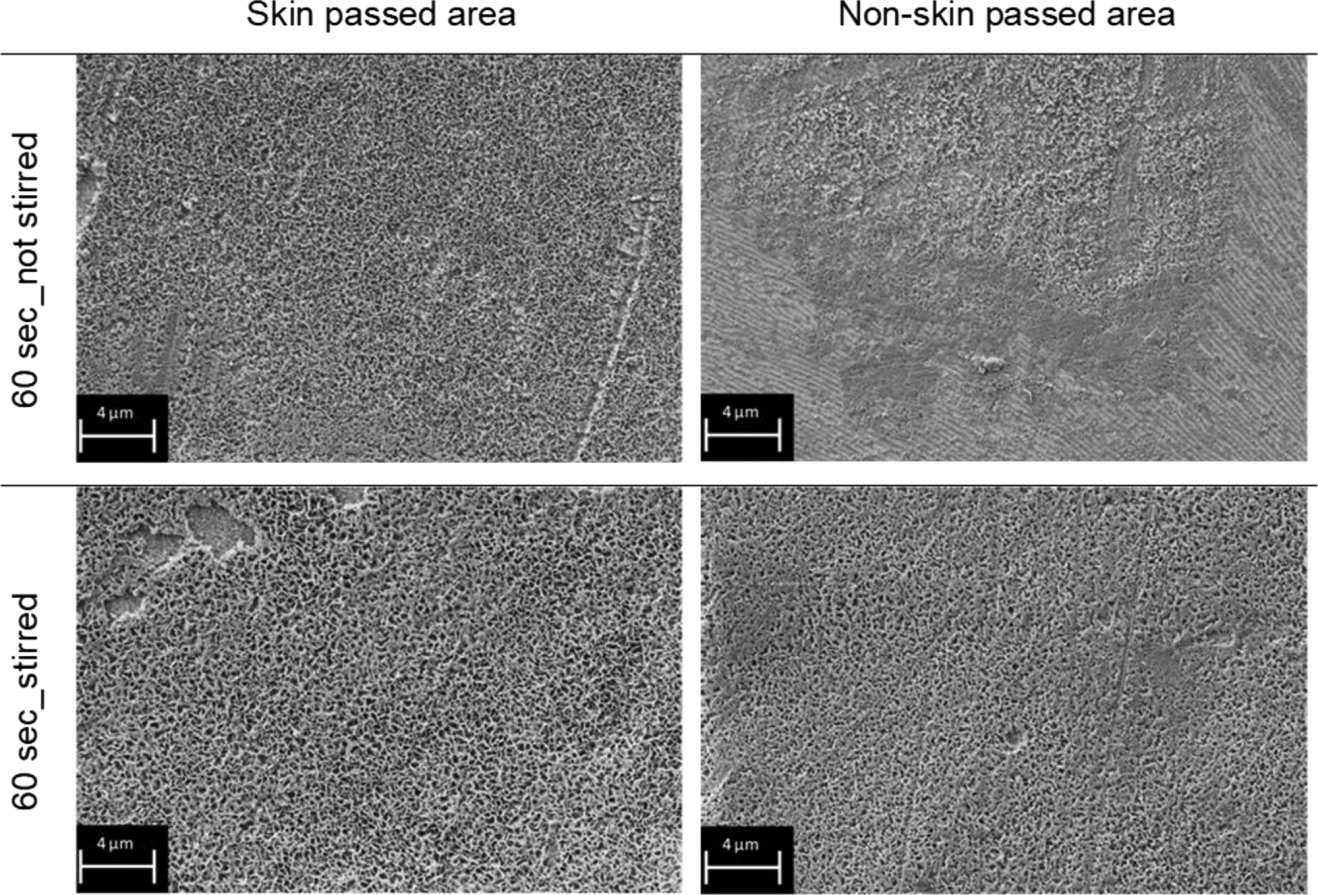
SEM images (top view) of samples immersed for 60 s into the stirred and nonstirred treatment solution. Representative images of the two different surface areas, namely, skin passed and non-skin passed area.

Figure 6 displays SEM images of the surface from ZM-coated steel samples that were immersed for 60 s into the stirred and nonstirred treatment solution. For each sample, a representative image of the two different surface conditions (see Section 2.1), namely, skin passed and non-skin passed areas, is shown.

On the skin passed area, both samples show a more or less fully covering LDH conversion layer, consisting of the typical hexagonal plate-like crystals standing vertically on the substrate and being connected with each other by the edges building a comb-like structure.^23,56−59^ However, on non-skin passed areas, only on the sample that was immersed into the stirred solution, a fully covering conversion layer was formed. Only a partial surface coverage with LDH crystals is present, when the treatment solution is not stirred.

Therefore, one can see again that the formation of an LDH conversion layer on ZM-coated steel is completed faster when there is a sufficient convection in the treatment solution, which we achieved by stirring the solution.

### 3.3 Influence of the Oxygen Concentration in the Treatment Solution on the LDH Conversion Layer Formation

In Section 3.2, we showed that the LDH conversion layer formation is accelerated by stirring the treatment solution. As discussed above, in nonstirred solution, a depletion of at least one reactant at the metal/solution interface leads to a diffusion-limited stage during conversion layer formation, which can be prevented by sufficient convection in the treatment solution. It is known that LDH compounds are possible corrosion products of ZM-coated steel,^46,60,61^ suggesting that oxygen plays an important role in the LDH conversion layer formation. Because the treatment solution has an alkaline pH value, oxygen reduction reaction will take place as the cathodic reaction during redox reactions and will be the driving force for the whole redox process, as commonly known for corrosion reactions.

We investigated the influence of oxygen in the treatment solution on the conversion layer formation with in situ OCP measurements using three different treatment solutions, that is, the standard solution, O_2_-enriched solution, and O_2_-depleted solution. The measurements were performed under stirred and nonstirred conditions for each solution.

**Figure 7:**
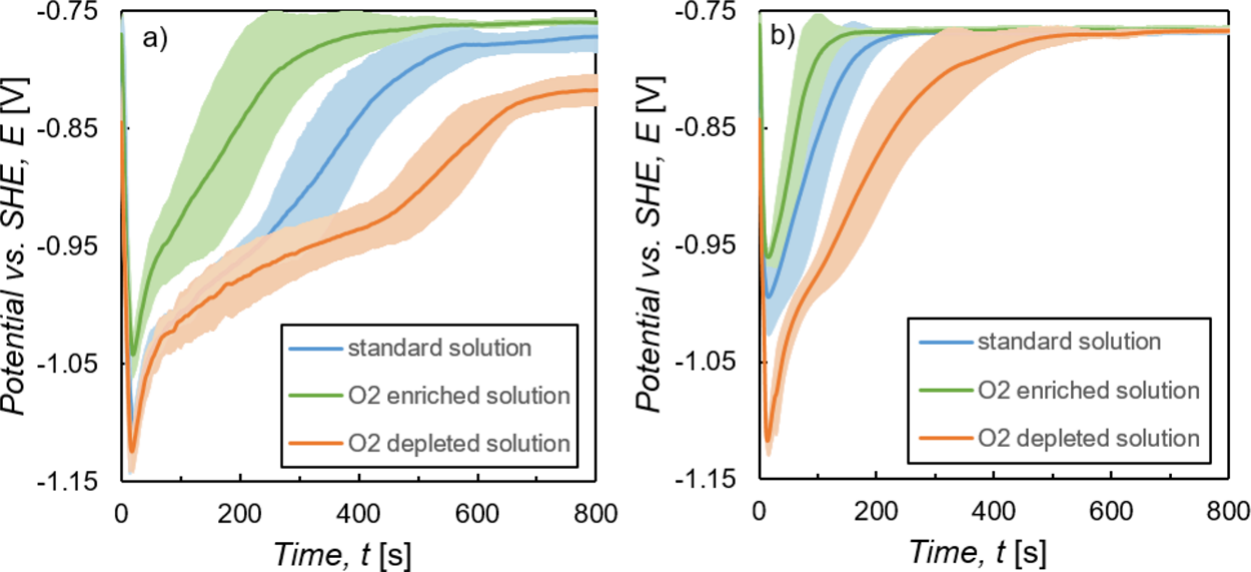
In situ OCP curves of ZM-coated steel immersed into the (a) nonstirred and (b) stirred standard (blue), O_2_-enriched (green), and O_2_-depleted (orange) solution. Average of four measurements with standard deviation.

Figure 7a shows the in situ OCP curves of ZM-coated steel samples immersed into the three different nonstirred treatment solutions. The shown graphs represent the average of four measurements each. With decreasing oxygen concentration, the plateau-like stage (see Section 3.2.1) during the potential rise gets more pronounced and lasts longer. Therefore, with lower oxygen concentrations, longer immersion times are necessary for the completion of the LDH conversion layer formation, indicated by reaching a constant potential.

The initially present reactants at the metal/solution interface are consumed during the underlying reactions, leading to the formation of LDH crystals on the surface. One of these reactions is the oxygen reduction leading to a depletion of oxygen at the interface, which will slow down the conversion layer formation. With an increased oxygen concentration in the treatment solution, the concentration gradient of oxygen between the metal/solution interface and the bulk solution increases, which in turn will lead to a higher diffusion flux of oxygen according to Fick’s first law (eq 1).
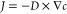
1

Therefore, a sufficient oxygen diffusion flux to the metal/solution interface will evolve faster, which results in a renewed rise of the slope, which can be seen clearly in Figure 7a. Thus, we can attribute the plateau-like stage in the in situ OCP curves in nonstirred solution to the depletion of oxygen at the metal/solution interface, which in turn indicates the importance of oxygen for the LDH conversion layer formation and that the underlying mechanism is in fact a corrosion reaction mechanism, consisting of reduction and oxidation reactions.

Figure 7b shows the in situ OCP curves of ZM-coated steel samples immersed into the three different stirred treatment solutions. The shown graphs represent again the average of four measurements each. As already described in Section 3.2.1, the in situ OCP curve in the stirred standard solution has no plateau-like stage or buckling during the potential rise. The same applies for the in situ OCP curve recorded in the O_2_ enriched solution. The constant potential is reached around 100 s faster in the O_2_-enriched solution compared to the standard solution. The in situ OCP curve during LDH layer formation in the O_2_-depleted solution has again a buckling in the potential rise and with roughly 500 s a much longer immersion time is needed to reach a constant potential, compared to the other two solutions. Also, one can see that in stirred solution, a decreasing oxygen concentration leads to an increasing immersion time necessary for the completion of the LDH conversion layer formation.

The rate-limiting factor for the LDH conversion layer formation on ZM-coated steel is the availability of oxygen at the metal/solution interface. The underlying reaction mechanism is a corrosion reaction mechanism, and therefore, the reduction reaction is the driving force of the overall reaction. Because the treatment solution is alkaline, oxygen reduction is the dominating reduction reaction. A sufficient oxygen availability at the metal/solution interface during the whole process of conversion layer formation is achieved by convection in the treatment solution. This leads to a more uniform oxygen distribution in the solution and a decrease in thickness of the diffusion-controlled layer between the metal and the bulk solution, consequently increasing the diffusion flux of oxygen. Higher oxygen concentrations in the treatment solution also lead to a higher oxygen availability and consequently to a faster completion of the LDH conversion layer formation.

### 3.4 Reaction Mechanism of the LDH Conversion Layer Formation

The results shown in Section 3.3 allowed us to conclude that the underlying reaction mechanism of the LDH conversion layer formation on ZM-coated steel is a corrosion reaction mechanism, like Persson et al. proposed for the formation of LDH compounds as corrosion products.^46^ The more noble phases of the ZM-coated steel, namely, the Al-phase and the Zn dendrites, will act as the cathode and the less noble MgZn_2_-phase as the anode.^46,62−64^ Because the treatment solution is alkaline, the dominant reduction reaction is the oxygen reduction, which will lead to a further alkalization at the cathodic sites of the sample. This further alkalization will lead to increased dissolution of Al as [Al(OH)_4_]^−^, which is referred to as cathodic dissolution of Al in the literature.^65−67^ The oxidation of the MgZn_2_ phase will lead to a dissolution of Mg and Zn, whereby the Mg dissolution takes place preferentially.^46,68,69^ Therefore, the Mg^2+^ concentration at the metal/solution interface will be considerably higher than the Zn^2+^ concentration. As a consequence of the described reactions, [Al(OH)_4_]^−^, Mg^2+^, OH^–^, and in small amounts Zn^2+^/[Zn(OH)_4_]^−^ will be present at the metal/solution interface. Additionally, there will be O_2_, Mg^2+^, OH^–^, and CO_3_^2–^, originating from the treatment solution itself. These ions will then lead to the formation/precipitation of the Mg/Al/CO_3_-LDH conversion layer on the ZM-coated steel, in terms of a corrosion product formation.

#### 3.4.1 Early Stage of the LDH Conversion Layer Formation

**Figure 8:**
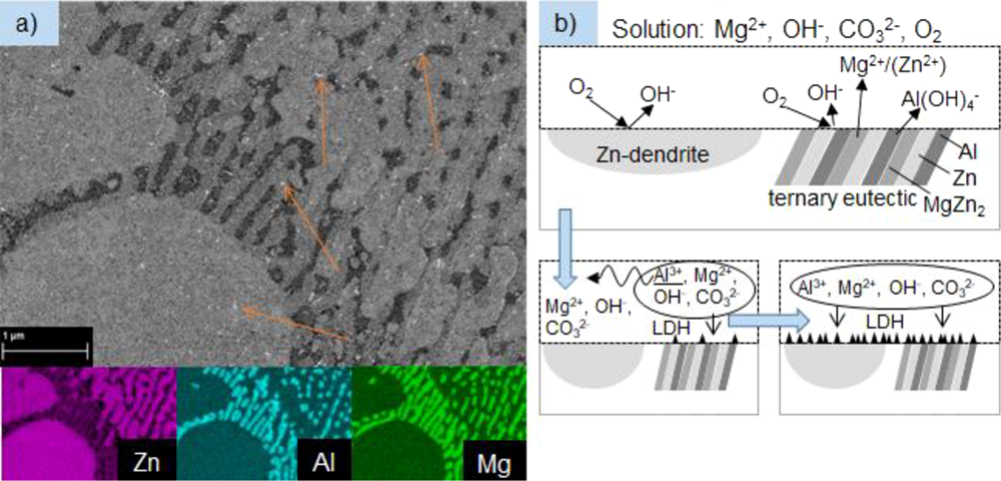
(a) SEM image (top view) of ZM-coated steel after 10 s immersion into the stirred treatment solution with EDX mappings of Zn (purple), Al (blue), and Mg (green). The beginning of crystals grown on the surface is exemplary marked with orange arrows. (b) Schematic diagram of the postulated mechanism of LDH crystal/–conversion layer formation on ZM-coated steel.

In Figure 8a, an SEM image of ZM-coated steel after immersion into the stirred treatment solution for 10 s is shown to investigate the early stage of the LDH formation. Additionally, EDX mappings of the shown area were recorded for Zn (purple), Al (blue), and Mg (green), which are also shown in Figure 8a. A schematic diagram of the abovementioned LDH conversion layer formation mechanism on ZM-coated steel is presented in Figure 8b, in which the insights into the early stage of LDH crystal formation described in this section are also considered.

After the immersion of the sample into the treatment solution for 10 s, small bright flake-like appearing depositions formed on the surface, which are exemplary marked by the orange arrows in the SEM image. One can see that in the area of the ternary eutectic (right side of the SEM image), significantly more of those depositions are present than on the Zn dendrites (left/bottom-left side of the image). Considering the XRD measurements shown in Section 3.4.3 and Figure 12, it can be assumed that these depositions are the beginning of LDH crystal formation on the surface because after 10 s, the reflex at 12.7° is already slightly visible. As described in Section 3.1, this reflex is assigned to an Mg–Al–CO_3_ LDH compound. A more detailed characterization of the compounds forming in the very early stage should be obtained in future work.

In the ZM coating, Al and Mg are exclusively present in the ternary eutectic, as can be seen in the EDX mappings also presented in Figure 8a. Therefore, the favored LDH crystal formation in the ternary eutectic compared to the Zn dendrites in the early stage of the conversion layer formation is in accordance with the above-stated mechanism. The reduction of oxygen can take place at the Zn dendrites and at the eutectic as well because at least one of the more noble phases Zn or Al is present in both. Mg^2+^, also needed for the LDH formation, is present in the treatment solution itself and therefore is available all over the sample as well, already at the metal/solution interface. However, the Al dissolution, increased through the stronger alkalization of the treatment solution due to the oxygen reduction, can only take place in the ternary eutectic. Consequently, in the very beginning of the LDH formation, all of the necessary ions for the LDH crystal formation, Al^3+^, Mg^2+^, OH^–^, and CO_3_^2–^, are present only in the areas of the ternary eutectic. This leads to an initial formation of LDH crystals on the ternary eutectic. With longer immersion times of the ZM-coated steel into the treatment solution, Al^3+^ is distributed more equally at the metal/solution interface, resulting in the LDH crystal formation on the Zn dendrites as well.

The exact role of the different components of the ternary eutectic, in terms of nucleation of the LDH crystals, and the possible pre-compounds forming on the surface before the formation of LDH crystals need to be explored further. Follow-up work for more detailed insights on the very early stage of the LDH formation on ZM-coated steel with more precise measuring techniques, for example, scanning Kelvin probe force microscopy or in situ XRD as used by Bouali et al.,^30^ needs to be conducted, which would exceed the scope of this study.

#### 3.4.2 Evolution of the Surface Coverage by the LDH Conversion Layer with Immersion Time

**Figure 9:**
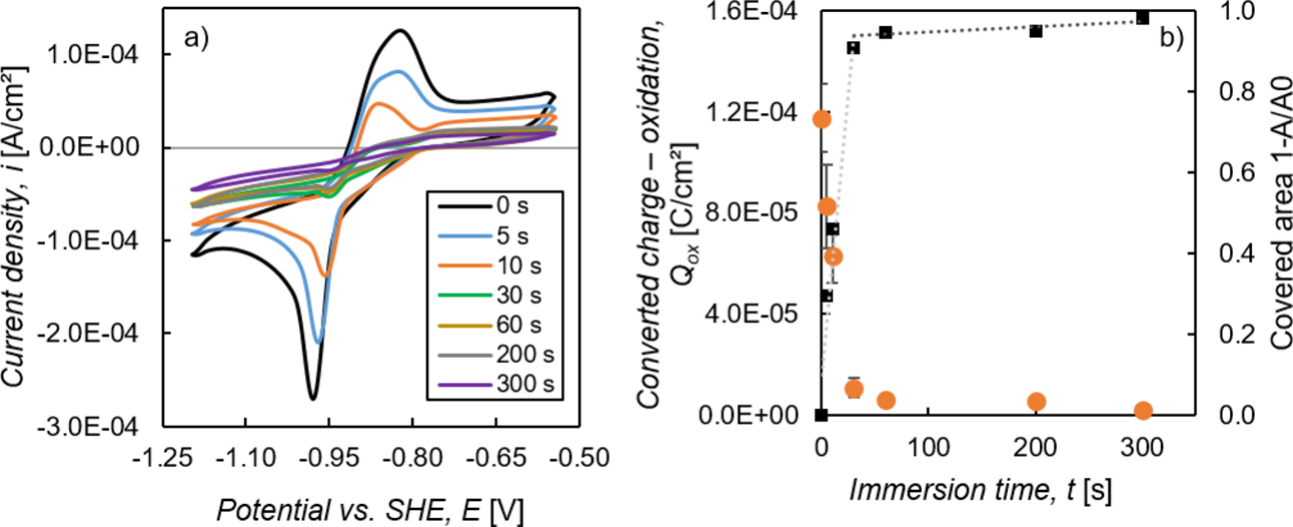
(a) Cyclic voltammograms (8th cycle) of samples immersed for 0–300 s into the stirred treatment solution. 0 s represents cleaned ZM-coated steel. (b) Converted charge during the oxidation reaction of the eighth cycle of the CV measurement (average of five measurements) and the calculated surface coverage with respect to the immersion time.

Figure 9a shows the cyclic voltammograms of samples immersed for 0, 5, 10, 30, 60, 200, and 300 s into the stirred treatment solution, whereby the 0 s sample represents cleaned ZM-coated steel. A decrease of both reduction and oxidation peaks with the increasing immersion time can be observed, indicating an increase of surface coverage by the formed LDH conversion layer.

As described in Section 2.3, the charge, which is converted during the oxidation reaction in the CV measurement *Q*_ox_, and the coverage of the ZM-coated steel substrate by the LDH conversion layer for different immersion times were calculated. In Figure 9b, these values are plotted against the different immersion times. After an immersion time of 30 s into the treatment solution, roughly 90% of the surface of the ZM-coated steel is covered with an LDH conversion layer. With longer immersion time, only a small additional increase of the coverage is achieved, and after 300 s, a coverage of around 98% is reached. Therefore, in the first 30 s of LDH conversion layer formation, more or less the whole surface of ZM-coated steel is covered by an LDH layer. It is likely that the conversion layer mainly grows in thickness with further immersion times, indicated by the slow increase of the coverage after 30 s immersion time.

**Figure 10:**
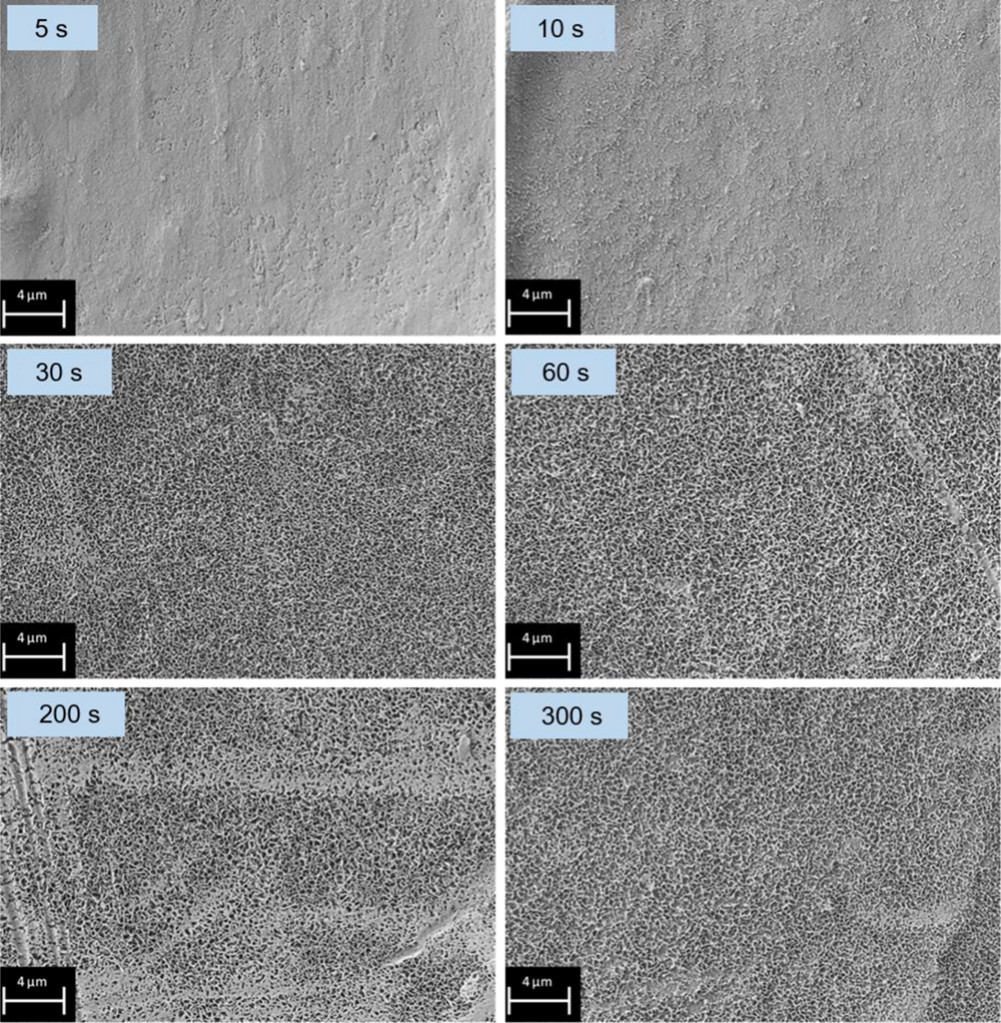
SEM images (top view) of samples immersed 5–300 s into the stirred treatment solution. Representative images only of skin passed areas are shown.

Figure 10 shows SEM images of the surface of ZM-coated steel after immersion into the treatment solution for 5, 10, 30, 60, 200, and 300 s. Only skin passed areas are shown for reasons of clarity, but no major differences, in terms of the LDH conversion layer, compared to the non-skin passed areas were present on the shown samples. After 5 s of immersion time, no pronounced LDH crystals are visible on the sample surface. An immersion time of 10 s leads to the formation of small crystal-like structures. After an LDH formation time of 30 s, a dense, homogeneous, and fully covering LDH conversion layer formed on the ZM-coated steel surface. The samples with longer immersion times do not differ from the 30 s sample, in terms of the optical appearance in the top view SEM images. Therefore, the top view SEM images indicate that during the first 30 s of conversion layer formation, a more or less fully covering LDH conversion layer is developed.

**Figure 11:**
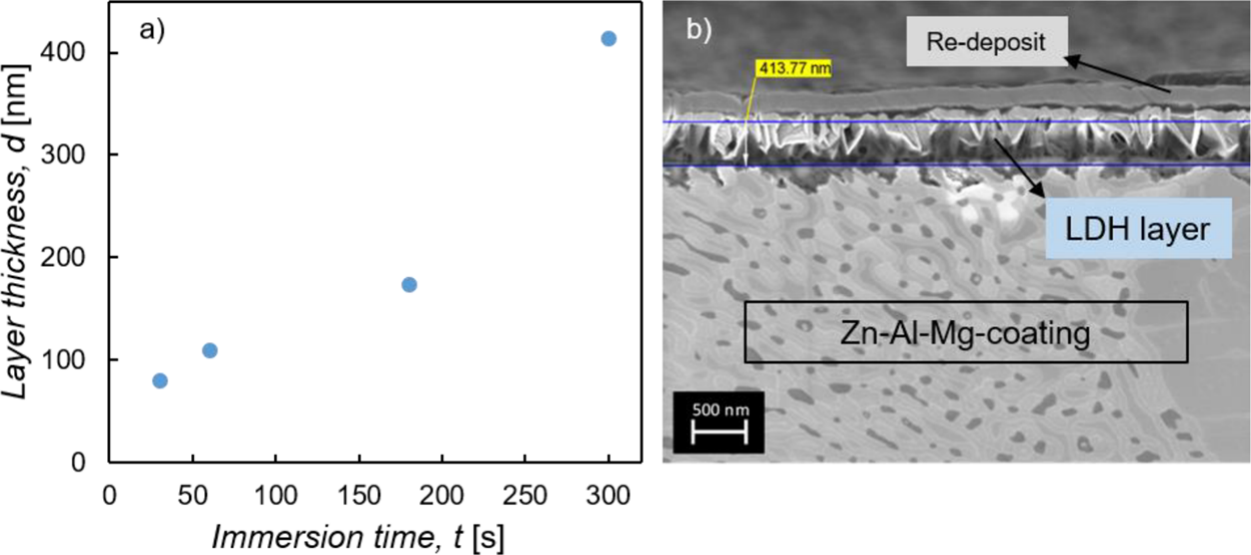
(a) LDH layer thickness of samples immersed for 30–300 s into the stirred treatment solution. (b) SEM cross-section view of the 300 s sample with measurement of the LDH layer thickness. A re-deposit layer deposits on top of the LDH layer during the cross-section polishing.

The thickness of the LDH layer with immersion times of 30, 60, 180, and 300 s were measured from cross-section view SEM images of the samples. In Figure 11a, the LDH layer thickness is plotted against the immersion time, and in Figure 11b, the cross-section SEM image of the sample with 300 s LDH formation time is shown.

After the surface is more or less fully covered with a LDH layer during the first 30 s of immersion time, as presented before, growth in layer thickness can be seen. An approximately linear growth within the examined immersion times was found.

#### 3.4.3 Growth of the LDH Conversion Layer Investigated by XRD Measurements

**Figure 12:**
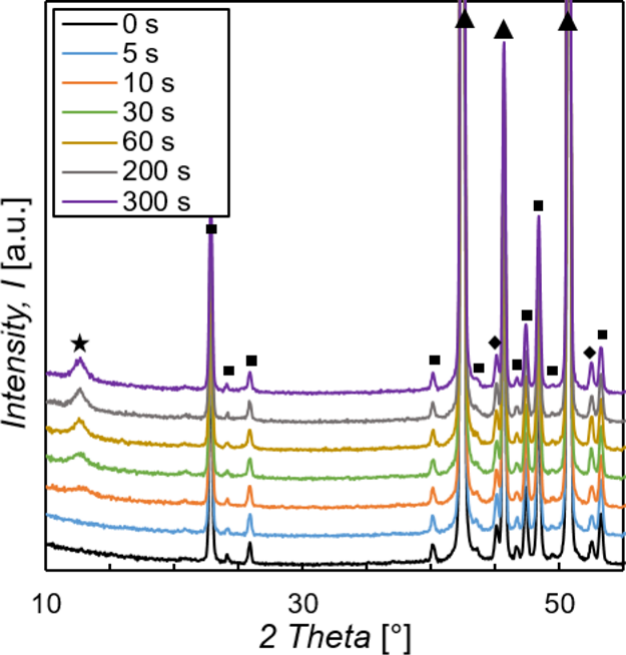
XRD patterns of samples immersed into the stirred treatment solution for 0–300 s in the 2θ range of 10–55°. 0 s immersion time represents the cleaned ZM-coated steel. Star, LDH; Squre, MgZn_2_; Triangle, Zn; Diamond, Al.

In Figure 12, the XRD patterns of ZM-coated steel samples after different times of immersion into the stirred treatment solution are shown in the 2θ range of 10–55°. At around 12.7°, a reflex begins to develop after 10 s immersion time. After 30 s, the reflex is already well marked, and after 60 s, no more changes in the reflex are noticeable. This reflex is characteristic for the 003 basal plane of LDH compounds and was assigned to an Mg–Al–CO_3_ LDH (Mg_0.833_Al_0.167_(OH)_2_(CO_3_)_0.083_·0.75H_2_O) compound by using the ICDD database.^57,24,70,71^ As mentioned in Section 3.1, the reflex at around 12.7° is the only change in the XRD pattern between ZM-coated steel without and with an LDH conversion layer. The additional reflexes of the LDH compound are not visible because of overlapping with much more intense reflexes of the phases that are present in the ZM coating, namely, Zn, Al, and MgZn_2_. These results are in good agreement with the results shown before in Section 3.4.2, indicating that the sample surface is more or less fully covered with the LDH conversion layer after around 30 s of immersion time and that first crystal growth is observable after 10 s.

### 3.5 Corrosion Protection

**Figure 13:**
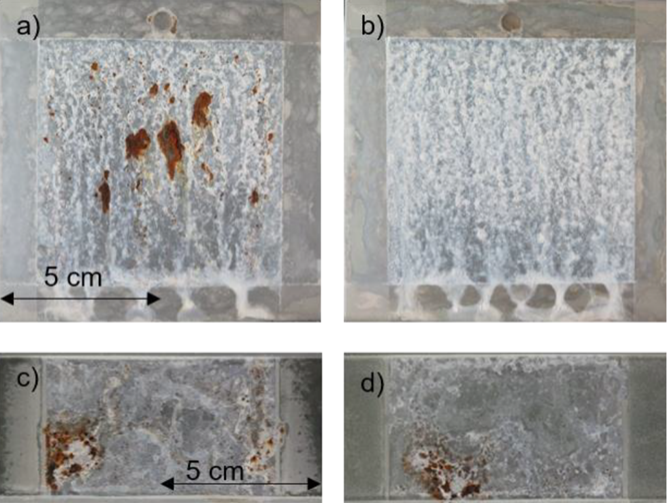
(a) ZM-coated steel after 2520 h in the NSS. (b) ZM-coated steel with an LDH layer (30 s formation time) after 2520 h in the NSS. (c) ZM-coated steel with a phosphate-based conversion layer after 4 cycles in the VDA 233-102 cyclic corrosion test. (b) ZM-coated steel with an LDH layer (3 min formation time) after 4 cycles in the VDA 233-102 cyclic corrosion test.

In Figure 13a,b, ZM-coated steel without and with an LDH conversion layer, after 30 s immersion into the treatment solution, is presented after 2520 h under NSS test conditions. One can see that the ZM-coated steel sample clearly shows red rust (corrosion products of the steel substrate under ZM coating), while on the sample with the LDH conversion layer, no red rust is visible. This shows an increased corrosion resistance of ZM-coated steel under NSS conditions due to the layered double hydroxide layer.

Figure 13c,d compares ZM-coated steel with an industrially used phosphate-based conversion layer with an LDH conversion layer (3 min formation time) after 4 cycles of the cyclic corrosion test VDA 233-102 according to DIN 55635. Both show a more or less equal coverage with red rust, and no major difference in the appearance of the two samples can be seen.

## 4 Conclusions

In this work, we studied the kinetics of LDH conversion layer formation on ZM-coated steel during immersion into a carbonate- and magnesium-containing alkaline solution. Stirring of the solution was found to increase the kinetics significantly. We demonstrated that the availability of oxygen at the metal/solution interface is essential for an effective LDH formation. As a result, in nonstirred solution, the diffusion-limited oxygen reduction reaction limits the overall LDH conversion layer formation, which can be prevented by high convection in the solution. As a consequence of the importance of oxygen on the conversion layer formation and the fact that LDH compounds are known as a possible corrosion product of ZM-coated steel, a corrosion-like reaction mechanism was proposed for the LDH conversion layer formation. A minimum immersion time of 30 s for obtaining a fully covering LDH conversion layer is necessary, while extended immersion times lead to a further growth in layer thickness. The deeper understanding of the kinetics and mechanism of the LDH conversion layer formation on ZM-coated steel, which was obtained through this work, gives rise to a targeted optimization of the treatment solution and process parameters, which is still necessary to ensure future applicability on continuous working industrial production plants.
